# Perioperative changes in cell-free DNA for patients undergoing surgery for colon cancer

**DOI:** 10.1186/s12876-022-02217-w

**Published:** 2022-04-06

**Authors:** Andreas W. Rosen, Mikail Gögenur, Isabella W. Paulsen, Jesper Olsen, Susanne Eiholm, Lene T. Kirkeby, Ole B. Pedersen, Niels Pallisgaard, Ismail Gögenur

**Affiliations:** 1 Center for Surgical Science, Department of Surgery, Zealand University Hospital, Lykkebækvej 1, 4600 Køge, Denmark; 2 Department of Clinical Immunology, Zealand University Hospital, Ringstedgade 77b, 4700 Næstved, Denmark; 3 Department of Science and Environment, Roskilde University, Universitetsvej 1, 4000 Roskilde, Denmark; 4 Department of Pathology, Zealand University Hospital, Sygehusvej 9, 4000 Roskilde, Denmark; 5 Department of Surgery, Zealand University Hospital, Sygehusvej 6, 4000 Roskilde, Denmark; 6 Faculty of Health and Medical Sciences, University of Copenhagen, Blegdamsvej 3B, 2200 Copenhagen N, Denmark; 7 Danish Colorectal Cancer Group, Copenhagen, Denmark

**Keywords:** Colon Cancer, Cell-free DNA, Perioperative, Surgery, Complications, Surgical stress

## Abstract

**Background:**

Various conditions with cellular decay are associated with elevated cell-free DNA (cfDNA). This study aimed to investigate if perioperatively measured cfDNA levels were associated with the surgical approach, complications, or recurrence.

**Methods:**

Plasma was obtained from patients who underwent surgery for colon cancer at admission and at the time of discharge. Quantitative measurement of cfDNA was performed by amplifying two amplicons of 102 base pairs (bp) and 132 bp of *Beta-2-Microglobulin* (*B2M*) and *Peptidyl-Prolyl cis–trans Isomerase A* (*PPIA*), respectively.

**Results:**

cfDNA was measured in 48 patients who underwent surgery for colonic cancer. Sixteen patients had recurrence during the follow-up period, fifteen developed a postoperative complication, and seventeen patients developed neither, acting as the control group. Postoperative cfDNA levels were significantly elevated from baseline samples, across all groups, with a median preoperatively *B2M* level of 48.3 alleles per mL and postoperatively of 220 alleles per mL and a median preoperatively level *PPIA* of 26.9 alleles per mL and postoperatively of 111.6 alleles per mL (*p* < 0.001 for *B2M* and *p* < 0.001 for *PPIA*). Postoperative levels of PPIA, but not B2M, were significantly higher in patients experiencing complications than in the control group (*p* = 0.036). However, a tendency towards an association between the surgical approach and the changes in cfDNA levels was found for PPIA (*p* = 0.058), and B2M (*p* = 0.087).

**Conclusions:**

Plasma cfDNA was increased after surgery in all patients with colon cancer. Postoperative PPIA levels were significantly higher in patients experiencing surgical complications but not in B2M levels.

## Background

Curative treatment of localized colorectal cancer (CRC) remains to be surgery. However, following curative surgery, patients with stage three cancer still have a thirty-three percent risk of recurrence, the leading cause of morbidity and mortality of CRC [1]. Surgery can also cause detrimental complications for the patients, with long-term side effects and readmissions, and prolonged time to adjuvant chemotherapy reducing the impact of the chemotherapy on oncological outcomes [2].

Identifying which patients are at high risk of recurrence in the perioperative period would enable targeting adjuvant chemotherapy at patients with an increased risk of recurrence, and patients with a low risk of recurrence could be spared of adjuvant chemotherapy. In addition, identifying patients at high risk of developing complications to surgery could enable targeted perioperative regimes with increased patient surveillance. Thus, an accurate biomarker of surgical stress that could predict if patients have an increased risk of complications or recurrence would be valuable.

Nucleic acids found in the plasma are a promising biomarker for the recurrence of disease in patients with malignant disease. They can be measured as cell-free DNA (cfDNA), found in various body fluids, such as stool, urine, cerebrospinal fluid, and pleural effusion [3–6]. Previous studies have shown that cfDNA levels are elevated in patients with CRC than healthy individuals [7]. A subset of cfDNA derived from mitochondria has been demonstrated to act as damage-associated molecular patterns [8]. This is of particular interest because the release of damage-associated molecular patterns helps promote post-traumatic immunosuppression [9]. This, in turn, potentially increases the risk of relapse for patients undergoing surgery for malignant diseases [10].

We have previously shown that acute trauma increases cfDNA concentration in blood and is correlated with mortality and severity of trauma [11]. We have also demonstrated that cfDNA increases following curative surgery for colorectal cancer [12]. In this exploratory study, we investigate if different surgical approaches, surgical complications, or later recurrence influenced perioperative cfDNA concentration in patients with colon cancer.

## Subjects and methods

### Patients

Repository samples from 48 patients undergoing surgery for colon cancer were analyzed in a case–control designed setting. The samples originated from patients admitted to the Department of Surgery of Zealand University Hospital, Denmark, with a histologically confirmed adenocarcinoma. The inclusion criteria were histologically confirmed adenocarcinoma, age ≥ 18, no prior radio- or chemotherapy, and written consent to participate in the study. All patients consented, and the inclusion period was between September 2006 and May 2012. During this period, laparoscopic surgery for colon cancer was introduced at the Department of Surgery at Zealand University Hospital, Denmark. In addition, standard postoperative care for the patients at the department included an enhanced recovery after surgery program.

All 48 patients had localized disease at the time of surgery. Sixteen patients had disease recurrence during the follow-up and no surgical complications. Fifteen patients developed surgical complications defined as Clavian-Dindo Class III or greater [13] but had no recurrence during the follow-up. A control group of seventeen patients with neither recurrence of disease during follow-up or surgical complications were selected from the cohort to match the event groups on variables sex, stage, age, and time to a blood test. Blood samples were drawn into EDTA tubes preoperatively the day before surgery and at the time of discharge from the ward. Samples were centrifuged once and stored in a − 80 degree freezer until analysis. cfDNA analysis was performed in July 2017 using ddPCR. Medical information was obtained from the patients’ medical records. Patients had a preoperative assessment with either a combined CT scan of the thorax and abdomen or a CT of the abdomen and a thorax X-ray. Patients were followed until recurrence, death from any cause, discontinuation in the postoperative follow-up program, or for a maximum of 5 years. The study was approved by the Danish National Committee on Biomedical Research Ethics (Protocol no.: Ø-2006-1-11G and SJ-373) and the Danish Data Protection Agency (approval no. 2014-41-2670).

### cfDNA

Plasma was stored at − 80 °C until analysis. cfDNA was isolated from 1 ml plasma on Magna Pure Compact Instrument (Roche Denmark, Denmark) using MagNA Pure Compact Nucleic Acid Isolation Kit I—Large Volume (cat.no.: 03730972001) according to manufacturer description. Droplet digital polymerase chain reaction (ddPCR) (QX200 instrument and reagents from Bio-Rad Laboratories, Inc. Hercules, CA, USA, droplet Generator Oil, Droplet Reader Oil, ddPCR Supermix) were used to measure genomic *Peptidyl-Prolyl cis–trans Isomerase A* (*PPIA)* (primer set from TAG Copenhagen A/S, probe from LGC Biosearch Technologies with 3′-BHQ1 and 5′-Fam) [14] and beta-2 microglobulin (*B2M*) (primer set from TAG Copenhagen A/S, probe from LGC Biosearch Technologies with 3′-BHQ1 and 5′-Fam) [15]. Primers and probes were combined in a multiplex ddPCR reaction and used to measure cfDNA. The DNA copy numbers obtained from ddPCR were converted to copy numbers/alleles per 1 ml plasma. Some samples did not contain sufficient plasma, and TE-buffer from Thermo Fisher Scientific cat.no.: 12090015 was added up to a total volume of 1000 µl. The dilution was corrected during the analysis. All samples were analyzed as duplicates with ddPCR.

### Statistical analysis

The mean of the ddPCR duplicates was used as the concentration of cfDNA. Due to the right-skewed distribution of cfDNA data non-parametric test was used to test the difference between the groups. A Wilcoxon signed-rank test was used for paired data between two groups, a Wilcoxon rank-sum test when testing for two groups with non-paired data, and a Kruskal Wallis rank-sum test when comparing more than two groups with non-paired data. The median and the corresponding 95% confidence interval (CI) of cfDNA concentration was calculated by bootstrapping with 5000 replications. The clinical parameters and visualization were analyzed using R (version 3.5.0, Vienna, Austria) and Rstudio (version 1.1.453, Boston, MA, USA), packages tidyr [16], dplyr [17] and plyr [18], for data manipulation. The package boot [19, 20] was used to calculate bootstrapped confidence intervals. The packages ggplot2 [21], cow plot [22], ggsinginf [23], ggpubr [24], and grid [25] were used for visualization. Lubridate [26] was used for calculations between dates. In this study, *p* values below 0.05 were considered significant.

## Results

We included 48 patients, of which 69% were male. Most patients had either stage II or III disease (46% and 48%). A laparoscopic procedure was the most common surgical approach (71%). In four patients, procedures started as laparoscopic but were converted to open surgery; in three of these, an open resection was considered more suitable, and in one of these, the change was done because of insufficient hemostasis during the laparoscopic procedure. The 48 patients were in three groups; patients who had a surgical complication, defined as a Clavien-Dindo classification of IIIb or greater (n = 15) [13], a recurrence in the study period (n = 16), or a control group without relapse or surgical complications (n = 17). The cohort had a median follow-up time of 48 months, ranging between 1 and 60 months. The end of the follow-up was January the 11th, 2017. Table 1 shows summarized patient demographics. A single patient had great variance between the concentration of B2M with the two preoperative measurements of 52.0 and 7680.0 alleles per mL plasma, respectively. The preoperative measurements of *PPIA* for the same patient were 24.4 and 28.0 alleles per mL plasma. The measurement of 7680 was notably above the range of other preoperative *B2M* observations. This was properly due to contamination with lymphocyte DNA during plasma preparation. Therefore, the sample was omitted for further analysis and replaced with the value of the other duplicate of 52.0 alleles per mL.

**Table 1:** Baseline characteristics

Group	Patients (48)		
	Complication (n = 15)	Recurrence (n = 16)	Control (n = 17)
Median Age (range), years	71 (53–82)	70 (56–88)	71 (46–87)
*Sex (%)*			
Male	12(80.0)	10 (62.5)	11 (64.7)
Female	3 (20.0)	6 (37.5)	6 (35.3)
*UICC stage (%)*			
I	2 (13.3)	0 (0.0)	1 (5.9)
II	10 (66.7)	3 (18.8)	9 (52.9)
III	3 (20)	13 (81.2)	7 (41.2)
Median time to postoperative blood sample (range), days	4 (2–10)	4 (1–18)	4 (2–14)
*Procedure (%)*			
Open	7 (46.7)	2 (12.5)	5 (29.4)
Laparoscopic	8 (53.3)	14 (87.5)	12 (70.6)

*UICC* Union of International Cancer Control

### No difference in preoperative cfDNA concentration

**Fig. 1:**
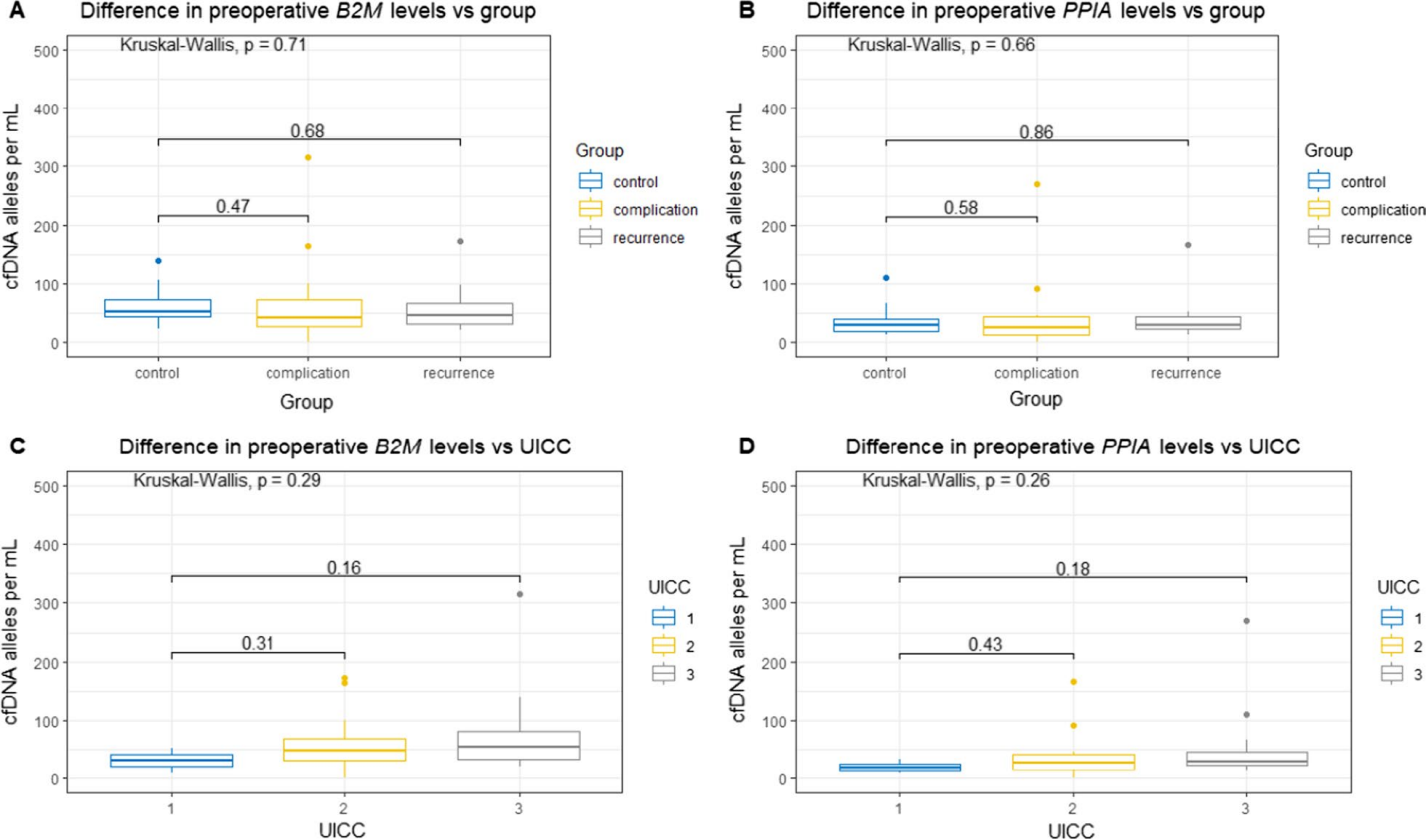
The difference in preoperative levels of PPIA and B2M, stratified for patient group and UICC. cfDNA levels depicted as boxplot showing median, upper and lower quartiles. Whiskers extend into a max of 1.5 times the interquartile range. Observations outside this are displayed as outliers. Wilcoxon rank-sum test was used for analyzing the difference between groups, Kruskal–Wallis test for difference across groups. **A** shows the difference in B2M levels across and in-between patient groups. **B** shows the difference in PPIA levels across and in-between patient groups. **C** and **D** depict the difference in B2M and PPIA levels stratified for the UICC stage. PPIA: Peptidyl-Prolyl cis–trans Isomerase A, B2M: Beta-2-microglobulin, cfDNA: cell-free DNA, UICC: Union for International Cancer Control

We found no difference in the preoperative cfDNA concentration across all groups (B2M: *p* = 0.71, PPIA: *p* = 0.66) or when comparing them individually with the control group (B2M: *p* = 0.47 and *p* = 0.68 for complication and recurrence groups, respectively. PPIA: *p* = 0.58 and *p* = 0.86 for complication and recurrence groups, respectively) (Fig. 1). No difference was likewise noted when stratifying for the UICC stage (B2M: *p* = 0.29, PPIA: *p* = 0.26).

### cfDNA concentration increases in postoperative samples

**Fig. 2:**
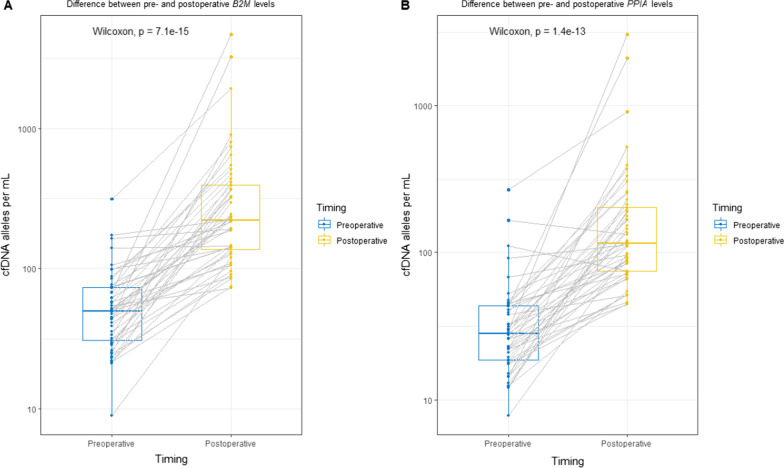
Change in cfDNA alleles per mL in pre-and postoperative samples. cfDNA levels depicted as boxplot showing median, upper and lower quartiles. Whiskers extend into a max of 1.5 times the interquartile range. Observations outside this are displayed as outliers. Y-axis scaled as log10. *p* values were calculated with Wilcoxon signed-rank test. **A** Shows the difference between B2M levels in pre-and postoperative samples. **B** Shows the difference between PPIA levels in pre-and postoperative samples. cfDNA: cell-free DNA, PPIA: Peptidyl-Prolyl cis–trans Isomerase A, B2M: Beta-2-microglobulin

The median concentration of *B2M* was 48.3 alleles per mL (95% CI 40.06–57.30 alleles per mL) preoperatively and 220 alleles per mL (95% CI 151.9–289.1 alleles per mL) postoperatively. For *PPIA,* the median concentrations were 26.9 alleles per mL (95% CI 21.71–32.05 alleles per mL) preoperatively and 111.6 alleles per mL (95% CI 71.0–145.4 alleles per mL) postoperatively (Fig. 2). cfDNA concentration was significantly elevated postoperatively for both *PPIA* and *B2M* in all groups (*p* < 0.001 for *B2M* and *p* < 0.001 for *PPIA*).

### Complications and surgical approach

**Fig. 3:**
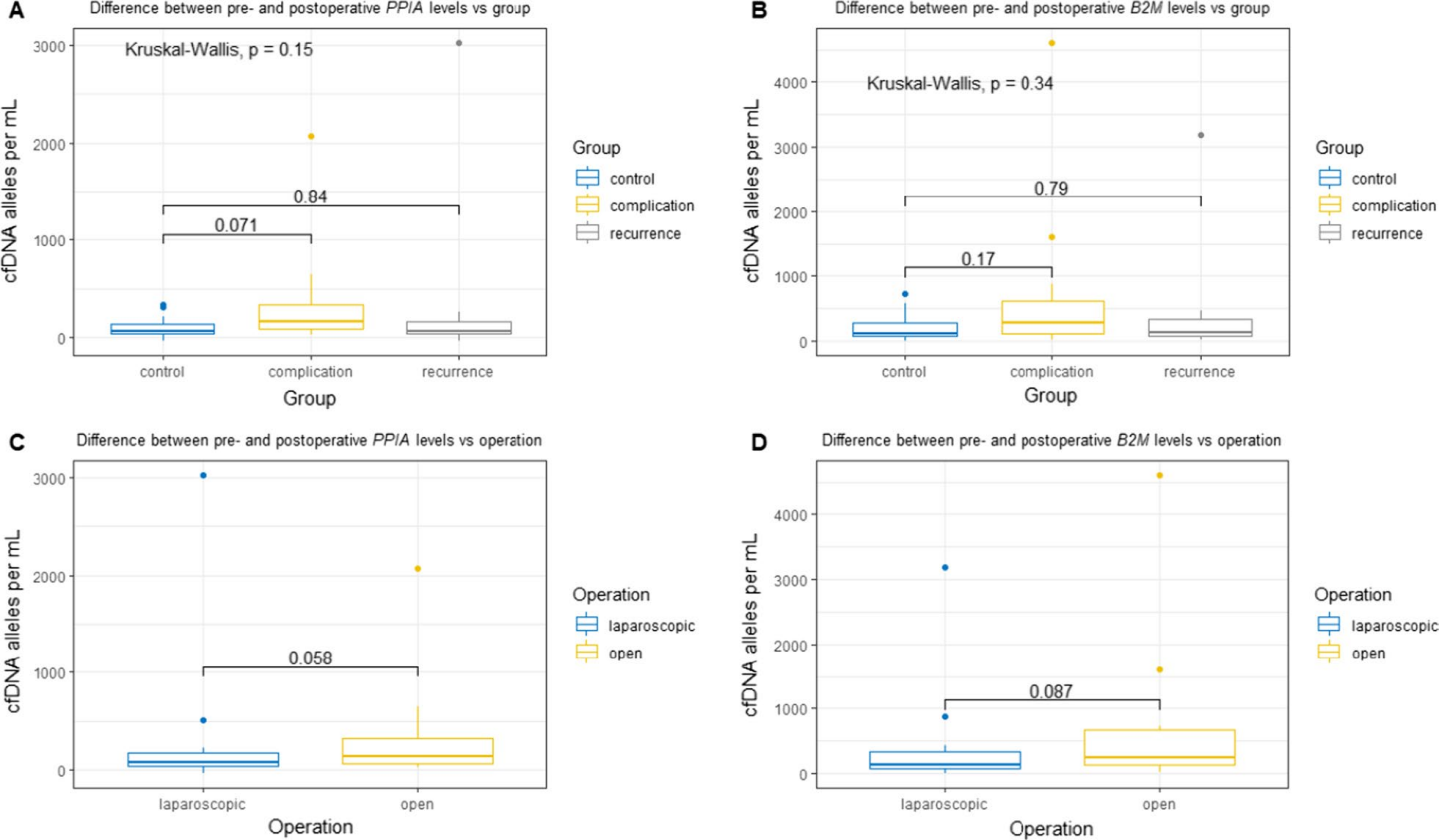
Change in cfDNA in alleles per mL vs. group and operation type. cfDNA levels depicted as boxplot showing median, upper and lower quartiles. Whiskers extend into a max of 1.5 times the interquartile range. Observations outside this are depicted as outliers. Wilcoxon rank-sum test was used for analyzing the difference between groups, Kruskal–Wallis test for difference across groups. **A** shows the relationship between changes in PPIA levels and patient group. **B** shows the relationship between changes in B2M levels and the patient group.** C** shows the relationship between changes in PPIA levels and operation procedure. **D** shows the relationship between changes in B2M levels and operation procedure. cfDNA: cell-free DNA, PPIA: Peptidyl-Prolyl cis–trans Isomerase A, B2M: Beta-2-microglobulin

When comparing the change of cfDNA in the perioperative period, we found a median change of 211.4 alleles *B2M* per mL (95% CI − 78.5 to 422.0) and 137.6 (95% CI 12.3–240.6) alleles *PPIA* per mL for the complication group, 144.7 alleles *B2M* per mL (95% CI − 10.2 to 251.4) and 65.2 alleles *PPIA* per mL (95% CI − 8.73 to 110.17) for the relapse group and 117.4 alleles *B2M* per mL (95% CI − 22.7 to 216.2) and 61.0 alleles *PPIA* per mL (95% CI 2.21–101.83) for the control group. No statistical difference between the groups was found for *PPIA* or *B2M* (*p* = 0.265 for *PPIA* and *p* = 0.497 for *B2M*), respectively. However, when comparing the groups individually, we noted a trend towards significance in PPIA levels for patients with complications (*p* = 0.071) (Fig. 3).

The median change in *B2M* concentration was 124.1 alleles per mL (95% CI 37.9–194.3) for patients who underwent a laparoscopic procedure and 246.1 alleles per mL (95% CI − 122.3 to 484.6) for patients who underwent an open procedure. The median change in *PPIA* was 71.1 alleles per mL (95% CI 31.30–107.56) for patients who underwent a laparoscopic procedure and 135.9 alleles per mL (95% CI − 43.5 to 252.5) for patients for underwent an open procedure. This showed that patients who underwent the open procedure had a borderline significantly elevated cfDNA concentration compared with patients who underwent a laparoscopic procedure (*p* = 0.058 for *PPIA* and *p* = 0.087 for *B2M*) (Fig. 3).

**Fig. 4:**
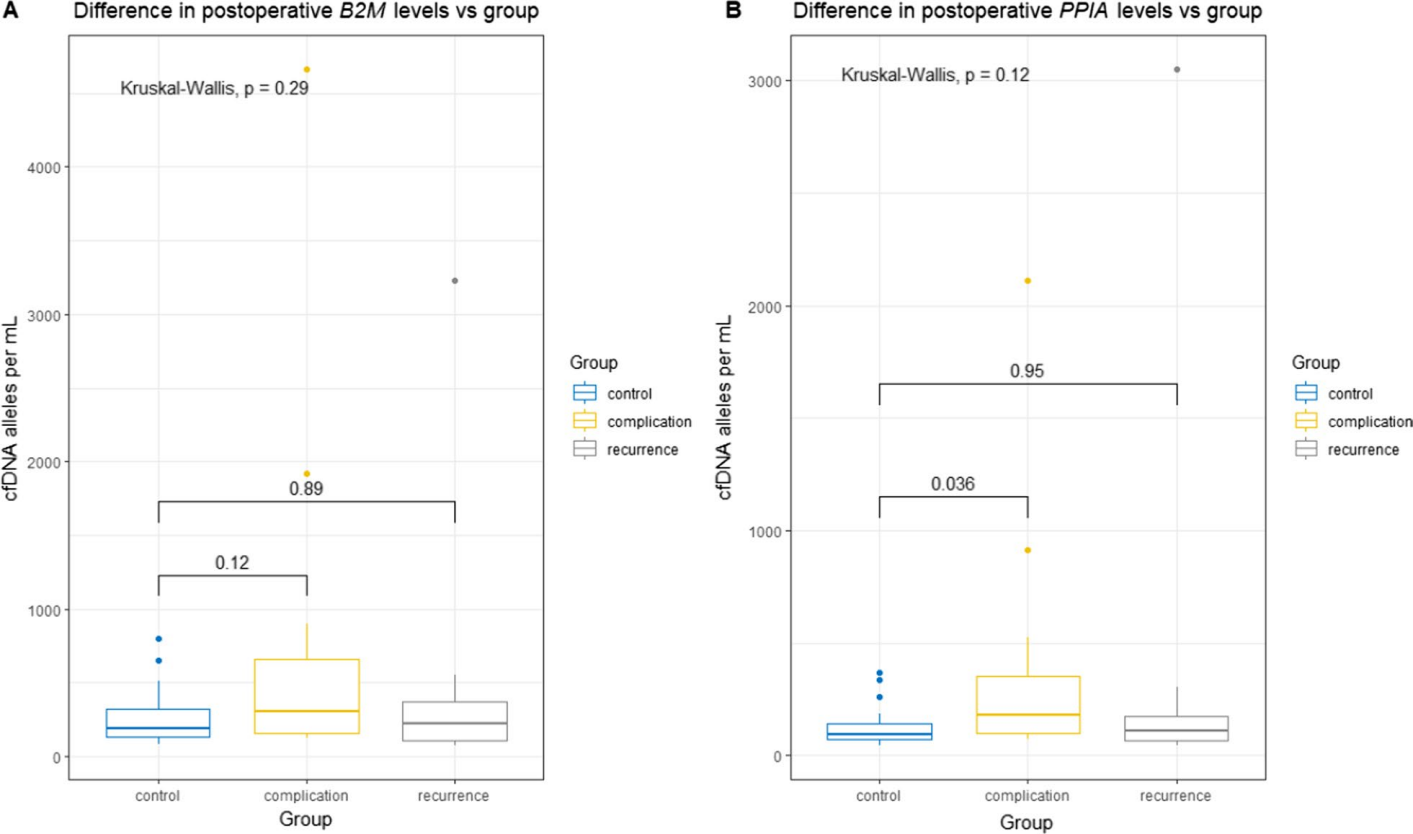
Difference between postoperative levels of B2M and PPIA stratified for the patient group. cfDNA levels depicted as boxplot showing median, upper and lower quartiles. Whiskers extend into a max of 1.5 times the interquartile range. Observations outside this are displayed as outliers. Wilcoxon rank-sum test was used for analyzing the difference between groups, Kruskal–Wallis test for difference across groups. **A** shows the difference in postoperative levels of B2M stratified for patient groups. **B** shows the difference in postoperative levels of PPIA stratified for patient groups. cfDNA: cell-free DNA, PPIA: Peptidyl-Prolyl cis–trans Isomerase A, B2M: Beta-2-microglobulin

When comparing postoperative levels of cfDNA, we noted that although there was no significant difference across all groups (*p* = 0.29 and *p* = 0.12 for B2M and PPIA levels, respectively), patients with complications had a significantly higher level of PPIA compared with controls (Fig. 4). No significance was noted for B2M levels.

## Discussion

The present study confirmed previous findings of elevation of cfDNA after surgical procedures in patients with colon cancer. In addition, we could show that the PPIA level after surgery was significantly elevated in patients with complications compared with controls.

Changes in the concentration of cfDNA following surgery have been described previously in a study that investigated the changes in cfDNA concentration perioperatively for patients who underwent liver transplantation either as a donor or recipient or patients with colorectal cancer [12, 27]. Similarly, a randomized clinical study investigated inflammatory markers in patients who underwent liver resection for liver metastases and randomized patients to either an open or laparoscopic procedure. Here the authors showed a significant increase in cfDNA in serial measurements as the area under the curve [28]. We have previously shown in a systematic review that acute trauma also induces increased cfDNA concentration [11]. With this study, we can confirm previous findings of the impact of surgery on cfDNA concentration and show a borderline significant difference between patients undergoing an open versus laparoscopic procedure. However, we could not show the influence of stage on cfDNA, which others previously have shown [29].

cfDNA is associated with inflammation [30], which might make cfDNA a viable marker to monitor surgical stress. Interestingly, we noted a significant difference between PPIA levels in patients experiencing surgical complications compared with controls.

In animal models, surgical stress is associated with increased tumor growth [31], making cfDNA a potential biomarker for surgical stress-induced tumor growth in some patients. In addition, a recent study showed that cfDNA was originating from the nucleus of cells corresponded with immunosuppression in trauma patients, while mitochondrial DNA did not [32]. This finding might explain some of the mechanisms seen in animal models between tumor growth and cfDNA.

However, we could not find a correlation between later development of recurrence and perioperative cfDNA.

A small study population limited this study which could explain why we could not confirm previous findings of higher cfDNA levels proportional with higher UICC stage. In addition, the distribution between patients undergoing open or laparoscopic surgery was different in the groups. Likewise, others have shown that age and comorbidities influence cfDNA concentration, which could impact our results [33]. The association between laparoscopic versus open surgery was thus only borderline significant, but we found a significant difference in PPIA levels for patients experiencing a surgical complication compared with controls. The difference between B2M and PPIA in our analysis could be affected by either gain or loss of PPIA or B2M alleles, which we previously have discovered in another study [34]. This data was not available for the current study.

## Conclusions

The present study found cfDNA to be statistically significantly elevated after surgery compared to the baseline. Even though a trend was evident in perioperative changes of cfDNA when comparing patients experiencing complications with controls, and a significant difference in postoperative PPIA levels, this study is limited due to its reduced number of patients. However, a borderline statistically significant association between changes in the cfDNA concentration and operation type was found. Further analyzing the implications of cfDNA on surgical stress with established markers of inflammation in more extensive studies are warranted.

## Data Availability

The datasets used and/or analysed during the current study are available from the corresponding author on reasonable request.

## Abbreviations

B2M: Beta-2 microglobulin
Bp: Base pairs
cfDNA: Cell-free DNA
CI: Confidence interval
CRC: Colorectal cancer
ddPCR: Droplet digital polymerase chain reaction
PPIA: Peptidyl-prolyl-cis–trans isomerase A
UICC: Union of International Cancer Control
